# Azithromycin decreases *Chlamydia pneumoniae*-mediated Interleukin-4 responses but not Immunoglobulin E responses

**DOI:** 10.1371/journal.pone.0234413

**Published:** 2020-06-08

**Authors:** Tamar A. Smith-Norowitz, Yvonne Huang, Jeffrey Loeffler, Elliot Klein, Yitzchok M. Norowitz, Margaret R. Hammerschlag, Rauno Joks, Stephan Kohlhoff

**Affiliations:** 1 Department of Pediatrics, Division of Infectious Diseases, State University of New York Health Sciences University, Brooklyn, New York, United States of America; 2 Department of Medicine, State University of New York Downstate Health Sciences University, Brooklyn, New York, United States of America; University of the Pacific, UNITED STATES

## Abstract

**Background:**

*Chlamydia pneumoniae* is an obligate intracellular bacterium that causes respiratory infection. There may exist an association between *C*. *pneumoniae*, asthma, and production of immunoglobulin (Ig) E responses *in vitro*. Interleukin (IL-4) is required for IgE production.

**Objective:**

We previously demonstrated that doxycycline suppresses *C*. *pneumoniae*-induced production of IgE and IL-4 responses in peripheral blood mononuclear cells (PBMC) from asthmatic subjects. Whereas macrolides have anti-chlamydial activity, their effect on *in vitro* anti-inflammatory (IgE) and IL-4 responses to *C*. *pneumoniae* have not been studied.

**Methods:**

PBMC from IgE- adult atopic subjects (N = 5) were infected +/- *C*. *pneumoniae* BAL69, +/- azithromycin (0.1, 1.0 ug/mL) for 10 days. IL-4 and IgE levels were determined in supernatants by ELISA. IL-4 and IgE were detected in supernatants of PBMC (day 10).

**Results:**

When azithromycin (0.1, 1.0 ug/ml) was added, IL-4 levels decreased. At low dose, IgE levels increased and at high dose, IgE levels decreased. When PBMC were infected with *C*. *pneumoniae*, both IL-4 and IgE levels decreased. Addition of azithromycin (0.1, 1.0 ug/mL) decreased IL-4 levels and had no effect on IgE levels.

**Conclusions:**

These findings indicate that azithromycin decreases IL-4 responses but has a bimodal effect on IgE responses in PBMC from atopic patients *in vitro*.

## Introduction

*Chlamydia pneumoniae* is an intracellular bacterium that infects humans and causes respiratory infections [1,2] in asthmatic and non-asthmatic subjects [2–5]. This bacterium activates immune cells (e.g. macrophages, epithelial cells); these cells produce cytokines that may contribute to asthma exacerbation [2]. In children with chronic respiratory disease, *C*. *pneumoniae* infection triggers the production of pathogen specific IgE, which may lead to inflammatory responses [6].

*C*. *pneumoniae*, can be treated with antibiotics (macrolides, tetracyclines, quinolones) that may have beneficial effects in patients with asthma [2,7]. Tetracyclines and macrolides have demonstrated anti-inflammatory activity independent of their antimicrobial activity [7,8].

Macrolides can affect the innate immune system [8]; alterations of pro-inflammatory cytokines (i.e. Interleukin (IL)-1beta, IL-8, IL-17) or tumor necrosis factor (TNF)-alpha have been described [8]. Azithromycin has been shown to inhibit activation of pro-inflammatory transcription factors in lung epithelial cells [9]. Bouwman, *et al*. reported that azithromycin inhibits production of IL-6, in hepatocytes infected with *C*. *pneumoniae* and cytomegalovirus [10].

Previous studies in our laboratory reported that treatment of asthmatic adults with minocycline had improvement in their asthma symptoms, independent of the presence of *C*. *pneumoniae* infection and may be due to past exposure [11]. This observed effect may be due to suppression of inflammation or eradication of the *C*. *pneumoniae* [3,12].

Other studies in our laboratory demonstrated that doxycycline suppressed *C*. *pneumoniae*-induced IgE and IL-4 responses in PBMC obtained from patients with asthma [13]. Smith-Norowitz *et al* recently reported that doxycycline suppressed *C*. *pneumoniae* induced interferon-gamma responses in PBMC in asthmatic children [14]. However, IL-4 levels did not significantly decrease after addition of ciprofloxacin (0.1 μg/ml) or azithromycin (1.0 μg/ml); *C*. *pneumoniae* infection and/or antibiotic treatment had no effect on IgE production *in vitro* [14].

The present study describes the *in vitro* effect of azithromycin on *C*. *pneumoniae* mediated IL-4 (Th2-type) cytokine responses and IgE responses in non-asthmatic atopic adults.

## Materials and methods

### Study design

Adult subjects (male/ female, 18 to 65 years old) were recruited from the outpatient department at SUNY Downstate Medical Center (Brooklyn, NY). Inclusion criteria included: non-asthmatic adult with atopy (defined by a single unequivocal positive skin test or history of atopic dermatitis or allergic rhinitis) without clinically defined persistent asthma symptoms [15], with low serum IgE levels (<100 IU/mL). Exclusion criteria included: history of chronic immunosuppressive or autoimmune disease, human immunodeficiency virus infection, cancer, antibiotic use, or immunotherapy, tobacco use within the past year, and incomplete follow-up. All subjects had a nasopharyngeal (NP) swab tested for *C*. *pneumoniae* and/or *M*. *pneumoniae* (determined by PCR), and peripheral blood (10mL) was collected. All clinical data was reviewed at the time of enrollment. The study was approved by the SUNY Downstate Medical Center Institutional Review Board (Brooklyn, NY). Written informed consent was obtained from all participants.

### Immunoglobulin determination: Total serum IgE

Total serum IgE levels were determined in serum using the UniCap Total IgE fluoroenzyme immunoassay (Pharmacia and Upjohn Diagnostics, Freiburg, Germany) as previously described [14]. Tests were performed in the Clinical Diagnostic Laboratory at SUNY Downstate Medical Center (Brooklyn, NY).

### Detection of C. pneumoniae-specific IgG antibodies

*C*. *pneumoniae*-specific IgG antibodies were measured using the microimmunofluorescence (MIF) test (AniLabsystems; Vantaa, Finland), as previously described [16].

### Preparation of *C*. *pneumoniae*

*C*. *pneumoniae* AR-39 (ATCC 53592; Manassas, VA) was propagated in HEp-2 cells as previously described [17].

### Cell cultures

PBMC were separated from blood on a Ficoll-Paque (GE Healthcare, Sweden) gradient (density 1.077) and put into cell culture a previously described [14], at 37°C in cRPMI medium in a humidified 5% CO_2_ atmosphere for up to 10 days. Cell viability was determined at 0, 48 and 240 hrs (>98%, 95%, and 90%, respectively), in the absence of any infection with *C*. *pneumoniae*.

### In vitro infection with *C*. *pneumoniae* and treatment with antibiotics

Following a 2 hr incubation to allow adherence, PBMC cultures were infected with *C*. *pneumoniae* (by adding purified EB for 1hr), or mock-infected (MI) and/or stimulated in the presence or absence of azithromycin (0.1 or 1.0 ug/mL) (Sigma) for either 48 hrs (IL-4) or up to 10 days (IgE) at 37°C in cRPMI in a humidified 5% CO_2_ atmosphere, as previously described [14]. All antibiotics were serially diluted (1:1, 1:2, 1:4, 1:10) [13] to determine optimal dose and kinetics [13], for suppression (for the purpose of cytokine production). Cytokine assays (IL-4) were run using supernatants collected from above cultures. The multiplicity of infection (MOI; 0.1) and time points (48h p.i. for cytokines ^10^ and 10d p.i. for IgE ^10^) used for analysis were selected by kinetic and dose response studies (using MOI of 0.01–10) for optimization of the assay. Two types of controls were used in infection experiments: identical volumes of heat-inactivated purified *C*. *pneumoniae* [13] and identical volumes of HEp-2 cell cultures not containing any bacteria processed the same way as the purified *C*. *pneumoniae* [17] based on dose-response experiments.

### Cytokine (IL-4) or IgE determination: ELISA

For the *in vitro* quantitative determination of human cytokine or IgE content in cell culture supernatants, solid-phase sandwich ELISA assays were performed using either cytokine (IL-4: IL-4 Human ELISA kit, Thermo Fisher Scientific, Waltham, MA) or IgE ELISA test kits (Bio Quant, San Diego, CA), according to the manufacturer’s recommended procedure, as previously described [14]. Cell culture supernatants were collected at either 48 hr p.i. (cytokines) [10] or 10 days p.i. (IgE) [10], by centrifugation, and samples were stored at -80° until analysis. Optical densities were converted to either IU/mL, ng/mL, or pg/ml (1 IU IgE = 2.4 ng/IgE protein). Detection limits for cytokine assay was: IL-4: <2.0 pg/mL.

### Quantitative real-time polymerase chain reaction (qPCR) of bacteria in swabs and cultures

Extractions of bacterial DNA from NP swab specimens [18] and PBMC were performed using a QIAAmp DNA Mini-Kit (Qiagen Inc., Valencia, CA), according to manufacturer’s recommendations. For PBMC cultures, supernatants (with adherent and non-adherent cells) were collected and bacterial DNA extracted. NP samples were stored in the freezer (-20°C), until analysis. DNA was extracted (QIAAmp DNA mini kit;Qiagen Inc), as previously described [18,19]. Specimens were tested for the presence and quantification of *C*. *pneumoniae* and *M*. *pneumoniae* DNA according to Apfalter et al. [18] and Waring et al. [19], using TAQMan technology-based qPCR (Light Cycler 2.0 platform; software version 4.0, Roche Diagnostics Corp, Indianapolis, IN). A specimen from either nostril positive for either *C*. *pneumoniae* or *M*. *pneumoniae* to represent a positive result.

### Statistical analysis

Data are expressed as means ± SD unless elsewise indicated. Data between cases were analyzed using the Mann-Whitney *U* test. A P value of <0.05 was considered significant. All statistical analyses were performed using Windows v.12.0 software (SPSS Inc., Chicago, IL).

## Results

### Study population demographics

Patient demographic and clinical characteristics is shown in Table 1. Five non- asthmatic atopic adult patients: 2 males (ages 24, 51) and 3 females (ages 51, 59, 65) were enrolled in this study. Total serum IgE levels were low (<100 IU/mL).

**Table 1:** Patient demographic and clinical characteristics.

Characteristic	Patient 1	Patient 2	Patient 3	Patient 4	Patient 5
**Age (y)**	24	51	51	59	65
**Gender**					
Female	(-)	(-)	(+)	(+)	(+)
Male	(+)	(+)	(-)	(-)	(-)
***C*. *pneumoniae* IgG Ab**	**(-)**	**(-)**	**(-)**	**(-)**	**(-)**
**Total serum IgE >100 IU/mL**	(-)	(-)	(-)	(-)	(-)
**Asthma**	**(-)**	**(-)**	**(-)**	**(-)**	**(-)**

Demographic data from non- asthmatic atopic subjects (N = 5). Serum IgE positive = >100 IU/mL

### Serological and nucleic acid amplification testing for *C*. *pneumoniae*

Non-asthmatic patients had negative *C*. *pneumoniae*-specific IgG MIF titers. All patients tested negative for *C*. *pneumoniae* and *M*. *pneumoniae* by qPCR (NP swabs).

### Effect of azithromycin on *C*. *pneumoniae*-induced IL-4 responses

**Fig 1:**
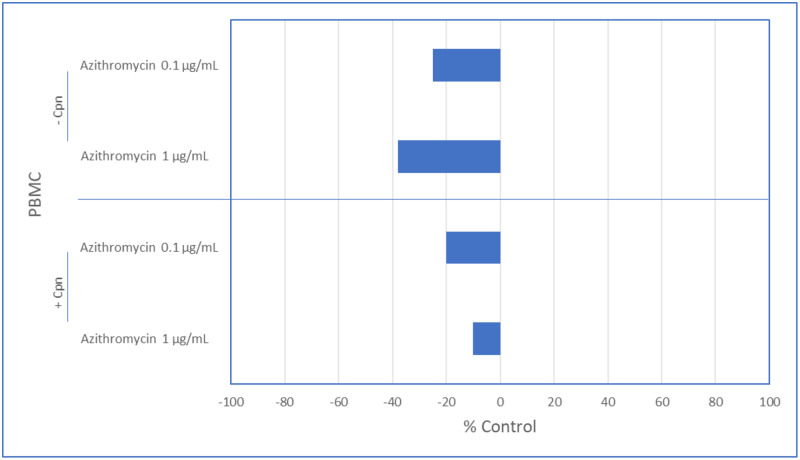
Effect of azithromycin on *Chlamydia pneumoniae* mediated IL-4 responses. Data represented as percent control. *P*>0.05, in all cases (Mann-Whitney U Test).

When azithromycin (0.1, 1.0 ug/mL) was added to cultures, IL-4 levels decreased (25%, 38%, respectively) (Fig 1). When PBMC were infected with *C*. *pneumoniae*, IL-4 levels decreased (17%). Addition of azithromycin (0.1, 1.0 ug/mL) decreased IL-4 levels (20%, 10%, respectively) (Fig 1). Thus, at low concentrations, azithromycin does modulate *C*. *pneumoniae*-induced production of IL-4.

### Effect of azithromycin on *C*. *pneumoniae-*induced IgE responses

**Fig 2:**
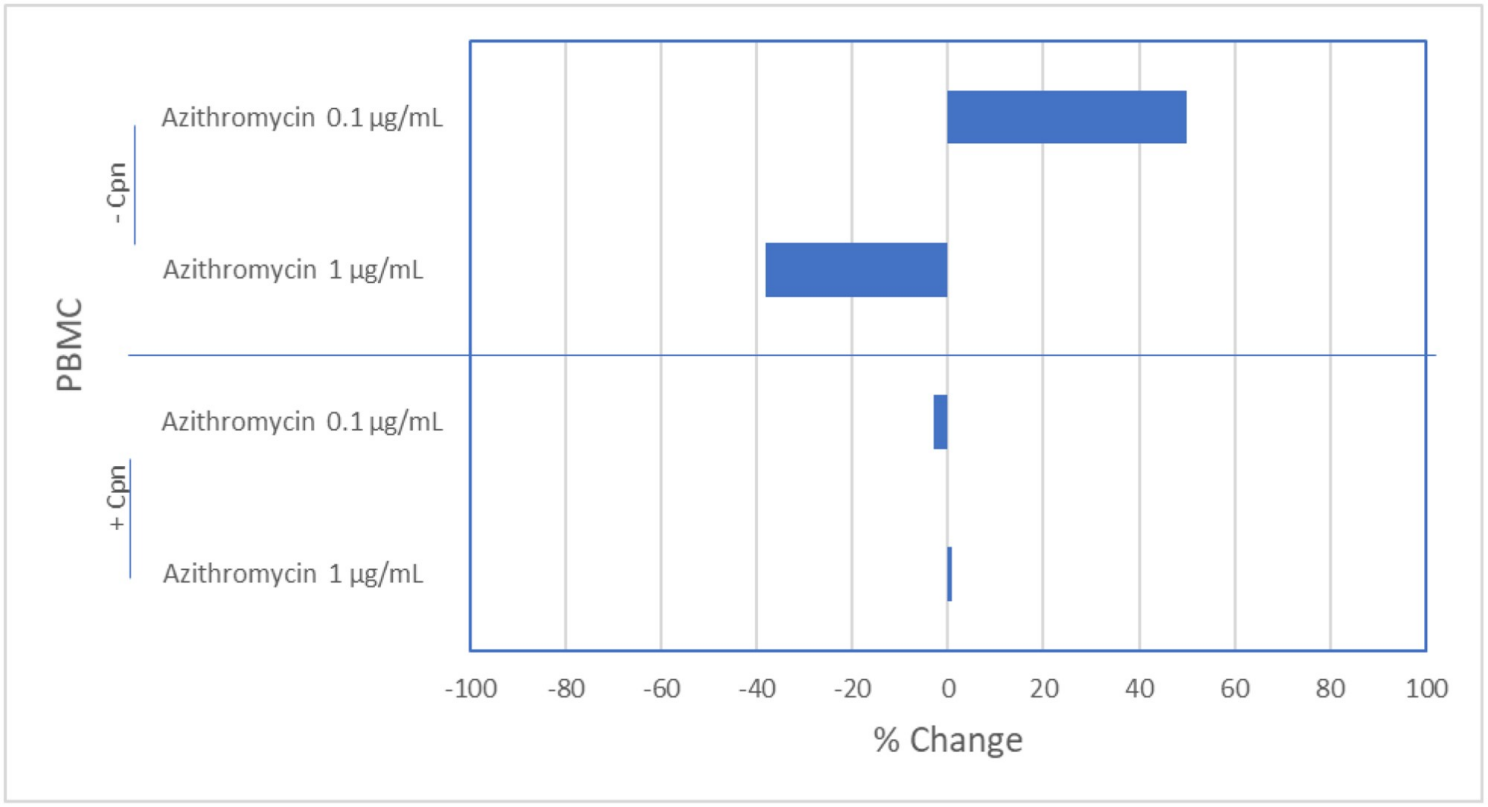
Effect of azithromycin on *Chlamydia pneumoniae* mediated IgE responses. Data represented as percent control. *P*>0.05, in all cases (Mann-Whitney U Test).

When azithromycin (0.1 ug/mL) was added to cultures, IgE levels increased (50%), but when 1.0 ug/mL was added IgE levels decreased (38%) (Fig 2). When PBMC were infected with *C*. *pneumoniae*, IgE levels decreased (63%). Addition of azithromycin (0.1, 1.0 ug/mL) had no effect on IgE levels (0–3%) (Fig 2). Thus, azithromycin does not modulate *C*. *pneumoniae*-induced production of IgE *in vitro*.

## Discussion

The effect of azithromycin on *in vitro C*. *pneumoniae* mediated cytokine and IgE responses and its contribution to inflammation are complex and not fully understood. In our initial report, we demonstrated that doxycycline suppresses *C*. *pneumoniae* mediated interferon-gamma responses in PBMC from children with asthma [14]; azithromycin did not decrease IL-4 or IgE responses [14]. The current study extends our prior study [14]; we further examine the *in vitro* effect of azithromycin on IL-4 and IgE response in *C*. *pneumoniae*-infected PBMC obtained from non-asthmatic adults. Successful treatment of *C*. *pneumoniae* infection with azithromycin may differ depending on asthma status.

Our first finding was that addition of azithromycin decreased IL-4 responses in *C*. *pneumoniae* infected PBMC from non-asthmatic subjects. Recent studies of Smith-Norowitz *et al* reported that doxycycline can suppress in vitro *C*. *pneumoniae* mediated interferon-gamma responses in asthmatic children [14]. However, addition of ciprofloxacin (0.1 μg/ml) or azithromycin (1.0 μg/ml) did not decrease IL-4 responses [14]; in vitro IgE production was unaffected [14]. Earlier studies reported from our laboratory showed that suppression of *C*. *pneumoniae*-induced cytokine and IgE responses by doxycycline in vitro was independent of anti-chlamydial activity in PBMC from asthmatic patients [13]. Those findings suggest that in patients with asthma, antibiotics have an important anti-inflammatory contribution [13]. The studies differ most probably due to other factors or components of inflammation in asthmatic patients. The findings indicate that the *in vitro* effect of azithromycin in PBMC differs depending on asthma status. Thus, the relevance of these properties may be different based on asthma status as well as atopy status and needs to be considered for treatment guidelines.

We also found that treatment of cells with azithromycin could not suppress *C*. *pneumoniae*-induced IgE production. The observed IgE production by azithromycin-treated infections might have been induced by other unknown contributors and not solely by *C*. *pneumoniae* infection. The IL-4 responses observed in our cultures represent a pathway for IgE production; it is possible that the failure of azithromycin to inhibit IgE responses was due to IL-4. The current findings are in agreement and supported by our previous studies [14] that reported azithromycin had no effect on *C*. *pneumoniae*-induced IgE responses in PBMC from patients with asthma [14].

The antibiotics azithromycin (macrolide), clarithromycin, and levofloxacin are used to treat *C*. *pneumoniae* respiratory infections in humans [20]. However, the recommended antibiotic doses may not be sufficient for *C*. *pneumoniae* infection eradication [20]. The current work extends our prior *in vitro* findings in asthmatic patients by examining the *in vitro* effect of azithromycin on *C*. *pneumoniae* mediated IL-4 and IgE responses in non-asthmatic atopic adults. Several important conclusions can be drawn from our data: (1) azithromycin decreases IL-4 responses but has a bimodal effect on IgE responses in PBMC from atopic patients *in vitro* and may have immunomodulatory properties; (2) at low concentrations, azithromycin does modulate *C*. *pneumoniae*-induced production of IL-4 but not IgE. Thus, depending on the concentration, azithromycin may inhibit production of proinflammatory mediators [10]; this may be independent of their antibacterial activities and due to the antimicrobiologic properties of azithromycin [10].

Some limitations to our study should be mentioned. We are aware of the small study number. Lastly, we did not elucidate the mechanism for the antibiotic effect on IL-4 production.

## Conclusions

These observations may have broad implications for the study of diseases such as *C*. *pneumoniae* in non-asthma and demonstrate the importance of disease-associated immune/inflammatory responses in non-asthmatic controls.

## References

[1] Hammerschlag MR, Kohlhoff SA, Gaydos CA. Chlamydia pneumoniae, in: Mandell G. L., Bennett J.E., Dolin R (Eds). Principles and Practice of Infectious Diseases, 8^th^ ed, Elsevier, Inc., Philadelphia, PA 2014, pp. 2174–82.

[2] Johnston SL, Martin RJ. *Chlamydophila pneumoniae* and *Mycoplasma pneumoniae* a role in asthma pathogenesis? Am J Respir Crit Care Med 2005; 172: 1078–89. 10.1164/rccm.200412-1743PP 15961690

[3] Emre U, Roblin PM, Gelling M, Dumornay W, Rao M, Hammerschlag MR, et al The association of *Chlamydia pneumoniae* infection and reactive airway disease in children. Arch Pediatr Adolesc Med 1994; 148: 727–31. 10.1001/archpedi.1994.02170070065013 8019629

[4] Hammerschlag MR, Chirgwin K, Roblin PM, Gelling M, Dumornay W, Mandel L, et al Persistent infection with *Chlamydia pneumoniae* following acute respiratory illness. Clin Infect Dis 1992; 14: 178–82. 10.1093/clinids/14.1.178 1571425

[5] Martin RJ, Kraft M, Chu HW, Berns EA, Cassell GH. A link between chronic asthma and chronic infection. J Allergy Clin Immunol 2001; 107: 595–01. 10.1067/mai.2001.113563 11295645

[6] Ikezawa S. Prevalence of *Chlamydia pneumoniae* in acute respiratory tract infection and detection of anti-*Chlamydia pneumoniae*-specific IgE in Japanese children with reactive airway disease. Kurume Med J 2001; 48(2): 165–70. 10.2739/kurumemedj.48.165 11501498

[7] Kohlhoff SA, Hammerschlag MR. Treatment of chlamydial infections: 2014 update. Expert Opin Pharmacother 2015; 16: 205–12. 10.1517/14656566.2015.999041 25579069

[8] Khan AA, Slifer TR, Araujo FG, Remington JS. Effect of clarithromycin and azithromycin on production of cytokines by human monocytes. Int J Antimicrobial Agents 1999; 11: 121–32. 10.1016/s0924-8579(98)00091-0 10221415

[9] Vanaudenaerde BM, Wuyts WA, Geudens N. Macrolides inhibit IL17-induced IL8 and 8-isoprostane release from human airway smooth muscle cells. Am J Transplant 2007; 7: 76–82. 10.1111/j.1600-6143.2006.01586.x 17061983

[10] Bouwman JJM, Visseren FLJ, Bouter PK, Diepersloot RJA. Azithromycin inhibits interleukin-6 but not fibrinogen production in hepatocytes infected with cytomegalovirus and chlamydia pneumoniae. J Lab Clin Med 2004; 144: 18–26. 10.1016/j.lab.2004.03.012 15252403

[11] Daoud A, Gloria CJ, Taningco G, Hammerschlag MR, Weiss S, Gelling M, et al Minocycline treatment results in reduced oral steroid requirements in adults. Asthma Allergy Asthma Proc 2008; 29: 286–94. 10.2500/aap.2008.29.3121 18534087

[12] Richeldi L, Ferrara G, Fabbri LM, Lasserson TJ, Gibson PG. Macrolides for chronic asthma. Cochrane database Syst Rev 2005; issue 4: CD002997. 10.1002/14651858.CD002997.pub2 16034882

[13] Dzhindzhikhashvili MS, Joks R, Smith-Norowitz TA, Durkin HG, Chotikanatis K, Estrella E, et al Doxycycline suppresses *Chlamydia pneumoniae*-mediated increases in ongoing immunoglobulin E and interleukin-4 responses by peripheral blood mononuclear cells of patients with allergic asthma. J Antimicrob Chemother 2013; 68: 2363–68. 10.1093/jac/dkt179 23749949

[14] Smith-Norowitz TA, Weaver D, Norowitz YM, Hammerschlag MR, Joks R, et al Doxycycline suppresses *Chlamydia pneumoniae* induced interferon gamma responses in peripheral blood mononuclear cells in children with allergic asthma. J Infect Chemother 2018; 24: 470–475. 10.1016/j.jiac.2018.02.004 29615379

[15] Cowen MK, Wakefield DB, Cloutier MM. Classifying asthma severity: objective versus subjective measures. J Asthma 2007; 44: 711–15. 10.1080/02770900701595576 17994399

[16] Wang SP, Grayston JT. Microimmunofluorescence serological studies with the TWAR organism In Oriel D, Ridgeway G eds. Chlamydial infections: Proceedings of the Sixth International Symposium on Human Chlamydial Infections. Cambridge: Cambridge University Press: 1986, 329–32.

[17] Roblin PM, Dumornay W, Hammerschlag MR. 1992. Use of HEp-2 cells for improved isolation and passage of *Chlamydia pneumoniae*. J Clin Microbiol 1992; 30: 1968–71. 1500500 10.1128/jcm.30.8.1968-1971.1992 PMC265424

[18] Apfalter P, Barousch W, Nehr M, Makristathis A, Willinger B, Rotter M, et al Comparison of a new quantitative *omp*A-based real-time PCR TaqMan assay for detection of *Chlamydia pneumoniae* DNA in respiratory specimens with four conventional PCR assays. J Clin Microbiol 2003; 41: 592–00. 10.1128/jcm.41.2.592-600.2003 12574252 PMC149699

[19] Waring AL, Halse TA, Csiza CK, Carlyn CJ, Arruda Musser K, Limberger RJ. Development of a genomics-based PCR assay for detection of *Mycoplasma pneumoniae* in a large outbreak in New York State. J Clin Microbiol 2001; 39: 1385–90. 10.1128/JCM.39.4.1385-1390.2001 11283060 PMC87943

[20] Kutlin A, Roblin PM, Hammerschlag MR. Effect of prolonged treatment with azithromycin, clarithromycin, or levofloxacin on *Chlamydia pneumoniae* in a continuous-infection model. Antimicrob Agents Chemother 2002; 46: 409–12. 10.1128/aac.46.2.409-412.2002 11796350 PMC127037

